# Aripiprazole once-monthly as maintenance treatment for bipolar I disorder: a 52-week, multicenter, open-label study

**DOI:** 10.1186/s40345-018-0122-z

**Published:** 2018-06-10

**Authors:** Joseph R. Calabrese, Na Jin, Brian Johnson, Pedro Such, Ross A. Baker, Jessica Madera, Peter Hertel, Jocelyn Ottinger, Joan Amatniek, Hiroaki Kawasaki

**Affiliations:** 1 University Hospitals Cleveland Medical Center, 10524 Euclid Avenue, Cleveland, OH 44106 USA; 2 Otsuka Pharmaceutical Development & Commercialization, Inc., Princeton, NJ USA; 3 H. Lundbeck A/S, Valby, Denmark; 4 Department of Psychiatry, Faculty of Medicine, Fukuoka University, Fukuoka, Japan

**Keywords:** Aripiprazole once-monthly, Bipolar I disorder, Maintenance treatment, Safety, Patient satisfaction

## Abstract

**Background:**

The long-acting injectable antipsychotic aripiprazole once-monthly 400 mg (AOM 400) was recently approved for maintenance treatment of bipolar I disorder (BP-I). The purpose of this study was to evaluate the safety, tolerability, and efficacy of AOM 400 as long-term maintenance treatment for BP-I.

**Methods:**

This open-label multicenter study evaluated the effectiveness of AOM 400 as maintenance treatment for BP-I by assessing safety and tolerability (primary objective) and efficacy (secondary objective). The study enrolled AOM 400-naive (“de novo”) patients as well as AOM 400-experienced (“rollover”) patients with BP-I from a lead-in randomized, placebo-controlled clinical trial that demonstrated the efficacy of AOM 400 in the maintenance treatment of BP-I (Calabrese et al. in J Clin Psychiatry 78:324–331, 2017). Safety variables included frequency and severity of treatment-emergent adverse events (TEAEs) and TEAEs resulting in study discontinuation. Efficacy was assessed by the proportion of patients maintaining stability throughout the maintenance phase, as well as mean changes from baseline in Young Mania Rating Scale (YMRS), Montgomery–Asberg Depression Rating Scale, and Clinical Global Impressions for Bipolar Disorder–Severity of Illness Scale (CGI-BP-S) total scores. Patient acceptability and tolerability of treatment was assessed using the Patient Satisfaction with Medication Questionnaire-Modified.

**Results:**

Of 464 patients entering the maintenance phase, 379 (82%) were de novo and 85 (18%) were rollover. TEAEs were more common in de novo than rollover patients. The overall discontinuation rate due to TEAEs was 10.3% (48/464). Improvements in YMRS and CGI-BP-S total scores were maintained during the study, and the vast majority of both de novo (87.0%) and rollover (97.6%) patients maintained stability through their last visit. Overall, the need for rescue medication during the maintenance phase was minimal (< 10% of patients). Patient satisfaction levels were high, with both de novo and rollover patients rating the side effect burden of AOM 400 as greatly improved relative to previous medications.

**Conclusion:**

AOM 400 was safe, effective, and well tolerated by both de novo and AOM 400-experienced patients with BP-I for long-term maintenance treatment.

*Trial registration* ClinicalTrials.gov, NCT01710709

**Electronic supplementary material:**

The online version of this article (10.1186/s40345-018-0122-z) contains supplementary material, which is available to authorized users.

## Background

Bipolar I disorder (BP-I) is a lifelong neuropsychiatric disorder characterized by manic episodes, and frequently includes depressive states intermixed with periods of remission. BP-I is specifically defined by periods of debilitating mania that impair social and occupational functioning and can include psychotic symptoms and the need for hospitalization (Grande et al. 2016). It has been shown that even during periods of remission, patients with BP-I exhibit high levels of functional and cognitive impairment, leading to a reduced quality of life (Martinez-Aran et al. 2004; Martino et al. 2009). Combined, these data suggest that treatment during both mood episodes and periods of remission is warranted.

Long-term pharmacologic therapy may be required to maintain periods of remission, prevent relapse of symptoms, and preserve function (Work Group on Bipolar Disorder 2002). The most commonly used medications for BP-I include mood stabilizers, anticonvulsants, and second-generation (atypical) antipsychotics (SGAs) (Grande et al. 2016). For patients with BP-I who experience severe episodes of mania, SGAs have frequently been used in conjunction with drugs such as lithium or valproate for stabilization during crises. Oftentimes these SGAs are then continued as adjunctive maintenance treatments (Lindström et al. 2017). More recently, certain SGAs have been approved for use as maintenance monotherapy, including risperidone and aripiprazole oral or bi-weekly and monthly formulations (Kishi et al. 2016; McIntyre et al. 2011). Oral therapy has traditionally been used for treatment of BP-I, but newer long-acting injectable (LAI) antipsychotic formulations may allow for better adherence rates, more consistent dosing, more regular contact between patients and their healthcare team, and improved patient outcomes (Brissos et al. 2014).

Though there are numerous oral options for maintenance treatment of BP-I, until recently, bimonthly risperidone LAI was the only LAI atypical antipsychotic approved by the US Food and Drug Administration (FDA) for maintenance treatment of BP-I (Risperdal Consta 2016). Randomized controlled trials have demonstrated the efficacy of bi-weekly risperidone LAI as monotherapy and adjunctive therapy in delaying time to recurrence of mood episodes in patients with BP-I (Macfadden et al. 2009; Quiroz et al. 2010). In an open-label trial involving a small study population (N = 11), patients on risperidone LAI maintenance treatment maintained symptom stability over a 12-month period (Han et al. 2007). Clinically relevant weight gain and glucose metabolism-related adverse events (AEs) have been more frequently reported in patients receiving risperidone LAI versus placebo (Quiroz et al. 2010). The recently approved once-monthly formulation of aripiprazole, aripiprazole once-monthly 400 mg (AOM 400), with its prolactin-sparing profile and low rate of sexual dysfunction side effects (Calabrese et al. 2017; Abilify Maintena 2016; De Hert et al. 2014), adds an additional treatment option for long-term maintenance treatment of BP-I.

Aripiprazole has also demonstrated a favorable metabolic side effect profile relative to other antipsychotic medications, which is important given that patients with BP-I exhibit increased risk for cardiometabolic disorders (Correll et al. 2015; Vancampfort et al. 2015). This bears even greater clinical significance considering that patients are typically required to take the medication long term for maintenance therapy. It has been shown that antipsychotic medications further exacerbate the risk for several cardiovascular and metabolic conditions beyond those experienced inherently by patients with bipolar disorder (Correll et al. 2015). Therefore, implementing the use of LAI antipsychotic medication formulations, which can make lower doses clinically effective (Kishi et al. 2016; Spanarello and Ferla 2014), should be a priority.

The primary and secondary objectives of the open-label trial reported here were to evaluate the safety/tolerability and efficacy, respectively, of AOM 400 for long-term maintenance treatment of BP-I in a mixed cohort of adult patients, some with no exposure to AOM 400 (de novo patients) and others stabilized and/or maintained on AOM 400 in the lead-in study (rollover patients). This is the first trial to study AOM 400 maintenance treatment in patients with BP-I not previously exposed to AOM 400 monotherapy and in those requiring rescue medication during maintenance treatment with AOM 400.

## Methods

### Study overview

This open-label, multicenter study (ClinicalTrials.gov, NCT01710709) was performed as a follow-on to a previous double-blind, placebo-controlled, randomized withdrawal study of AOM 400 monotherapy in the maintenance treatment of BP-I, as previously published (Calabrese et al. 2017). The study reported here enrolled both rollover patients from the aforementioned study and AOM 400-naive (de novo) patients to assess the long-term safety and efficacy of AOM 400 during ≥ 1 year of maintenance treatment in patients with BP-I. Unlike the patients rolling over from the lead-in study, de novo patients were not stabilized on AOM 400 before the maintenance phase (although they were required to be stable on oral aripiprazole to enter the study), and use of adjunct rescue medications was permitted.

This trial was conducted from November 2012 to November 2016 at 149 sites in 10 countries (Canada, France, Hungary, Japan, Malaysia, Poland, Romania, South Korea, Taiwan, and the United States) in compliance with the International Conference of Harmonisation and Good Clinical Practice consolidated guideline (2016). The protocol was approved by an institutional review board or independent ethics committee, as appropriate. Informed consent was obtained from all patients or their legal representatives as necessary.

Safety was assessed by the frequency, severity, seriousness, and discontinuation of AOM 400 due to treatment-emergent adverse events (TEAEs). Efficacy was assessed by (a) the proportion of patients meeting stabilization criteria at the beginning of the maintenance phase who remained stable at the last visit; (b) mean changes from baseline in Young Mania Rating Scale (YMRS) (Young et al. 1978) and Montgomery–Asberg Depression Rating Scale (MADRS) (Montgomery and Asberg 1979) total scores, as well as in Clinical Global Impressions for Bipolar Disorder-Severity of Illness Scale (CGI-BP-S) (Spearing et al. 1997) scores (overall, mania, and depression); and (c) proportion of patients requiring use of a rescue medication, as well as the frequency and duration of use of the rescue medication. Patient acceptability and tolerability of treatment was assessed by (a) patient satisfaction with AOM 400 and (b) side effects of AOM 400 versus previous medication, both measured using the Patient Satisfaction with Medication Questionnaire–Modified (PSMQ-M) (Kalali 1999).

### Patients

This study enrolled 2 cohorts of patients, defined as de novo or rollover. De novo patients consisted of male and female outpatients aged 18–65 years with a diagnosis of BP-I according to *Diagnostic and Statistical Manual of Mental Disorders, Fourth Edition, Text Revision* (American Psychiatric Association 2000) criteria, and confirmed by the Mini-International Neuropsychiatric Interview (Sheehan et al. 1998). Eligible patients had experienced ≥ 1 previous manic or mixed episode of sufficient severity to require hospitalization, treatment with a mood stabilizer, or treatment with an antipsychotic agent. Patients with a current depressive episode or a history of ≥ 9 episodes in the past year were excluded. Eligibility criteria for this study were generally similar to those of the lead-in study (Calabrese et al. 2017) except that the lead-in study excluded patients with either a mixed or depressive episode, whereas the present study excluded only patients experiencing a depressive episode. Additionally, there was no YMRS score criterion for entry into this open-label study.

Rollover patients were derived from the lead-in double-blind, placebo-controlled randomized withdrawal trial as previously published (Calabrese et al. 2017). Patients were required to have completed the maintenance phase without recurrence to be eligible for this extension study. Because the final phase of the lead-in trial was randomized, patients entering the current study were from both the placebo and AOM 400 maintenance arms, resulting in patients with different levels of AOM 400 exposure before inclusion in the current study. However, all rollover patients had some level of AOM 400 exposure owing to the 12- to 28-week AOM 400 stabilization phase included as part of the lead-in trial protocol (Calabrese et al. 2017).

### Study design

This trial had distinct protocols for de novo versus rollover patients. For de novo patients, the study consisted of 2 or 3 of the following phases: a 4- to 6-week oral aripiprazole cross-titration phase, if the patient was receiving an oral antipsychotic other than aripiprazole at enrollment, a 4- to 12-week oral aripiprazole stabilization phase, and an open-label 52-week maintenance phase where AOM 400 was administered by single-site gluteal intramuscular injection every 4 weeks. Of note, no stabilization on AOM 400 was performed before the AOM 400 maintenance phase. De novo patients entered the study at screening and then proceeded to oral conversion or oral stabilization, depending on their current antipsychotic treatment regimen.

Because cross-titration and stabilization were completed as part of the lead-in trial, rollover patients entered this study directly at the AOM 400 maintenance phase (Calabrese et al. 2017). Importantly, only those patients completing the lead-in study through the full 52-week maintenance phase without recurrence of a mood episode were eligible for participation in the current study. In both de novo and rollover patients, dose decrease to 300 mg was permitted for tolerability, as was return to the 400-mg dose at any point.

Use of rescue medications was permitted if a patient became unstable. Patients requiring rescue therapy were permitted use of one adjunct medication at a time, as follows: lithium or valproate (immediate release or controlled release), benzodiazepines (lorazepam equivalents) at ≤ 4 mg/day (≤ 12 mg/week) during the oral aripiprazole stabilization phase, and ≤ 2 mg/day (≤ 8 mg/week) during the AOM 400 maintenance phase.

### Stability assessments

Before entering the AOM 400 maintenance phase, de novo patients were required to meet the following stability criteria: outpatient status, YMRS total score ≤ 12, MADRS total score ≤ 12, and no active suicidality, with active suicidality defined as MADRS item 10 score ≥ 4, or “yes” on question 4 or 5 of the Columbia Suicide Severity Rating Scale (C-SSRS) (Posner et al. 2009).

### Safety and tolerability

All patients who received ≥ 1 dose of AOM 400 in the open-label maintenance phase were analyzed for safety. Safety data were summarized using descriptive statistics for the open-label AOM 400 maintenance phase. AEs were coded according to Medical Dictionary for Regulatory Activities definitions. A TEAE was defined as an AE that emerged after the start of treatment or an AE that continued from baseline of one phase and became serious; was drug related; or resulted in death, discontinuation, interruption, or reduction of dosing during the subsequent phase. Suicidality was measured using the C-SSRS. Injection site reaction was assessed using the patient-reported Visual Analog Scale (VAS) and investigator evaluations. Extrapyramidal symptoms were reported as the change from baseline (beginning of AOM 400 maintenance phase) in Simpson–Angus Scale (used in countries other than Japan) (Simpson and Angus 1970), Drug-Induced Extrapyramidal Symptoms Scale (used in Japan) (Kim et al. 2002), Abnormal Involuntary Movement Scale (Guy 1976), and Barnes Akathisia Rating Scale (Barnes 1989) scores. Standard safety measurements, including clinical laboratory tests (including serum prolactin concentrations), vital signs, electrocardiogram (ECG), body weight, and general physical examination, were recorded. Central laboratories designated by the sponsor were used for all laboratory testing and ECG review.

### Efficacy

All patients with ≥ 1 postbaseline effectiveness evaluation in the open-label AOM 400 maintenance phase were analyzed for efficacy. The proportion of patients stable at baseline who were also stable at the last visit of the maintenance phase was calculated using descriptive statistics. The mean changes from baseline to end of study (up to week 52) in YMRS and MADRS total scores and in CGI-BP-S overall score were calculated using descriptive statistics with the last-observation-carried-forward method.

Briefly, the YMRS is based on a clinical interview with patients in which they report on 11 items that assess the core symptoms of mania. Total score can range from 0 to 60, with higher scores indicating more severe mania (Young et al. 1978). The MADRS is a structured interview that assesses the patient’s depressive symptoms. Using a scale consisting of 10 items scored on 7 defined grades of severity (0–6), the total score can range from 0 to 60, with higher scores indicating greater severity of depressive symptoms (Montgomery and Asberg 1979). Finally, the CGI-BP-S is a 7-point scale that rates a patient’s severity of bipolar illness in 3 categories: mania, depression, and overall; higher scores indicate more severe disease (Spearing et al. 1997). All raters of efficacy assessments received structured training.

### Patient satisfaction and patient-assessed tolerability

The PSMQ-M (Kalali 1999) was used to evaluate patient satisfaction with AOM 400 and to determine the side effect burden of AOM 400 relative to the patient’s previous medications. These data were interpreted as patient-assessed tolerability of AOM 400 as maintenance therapy for BP-I. The PSMQ-M is a simple 4-part questionnaire that patients complete independently, requiring little to no clinician aid.

## Results

### Patients

**Fig. 1:**
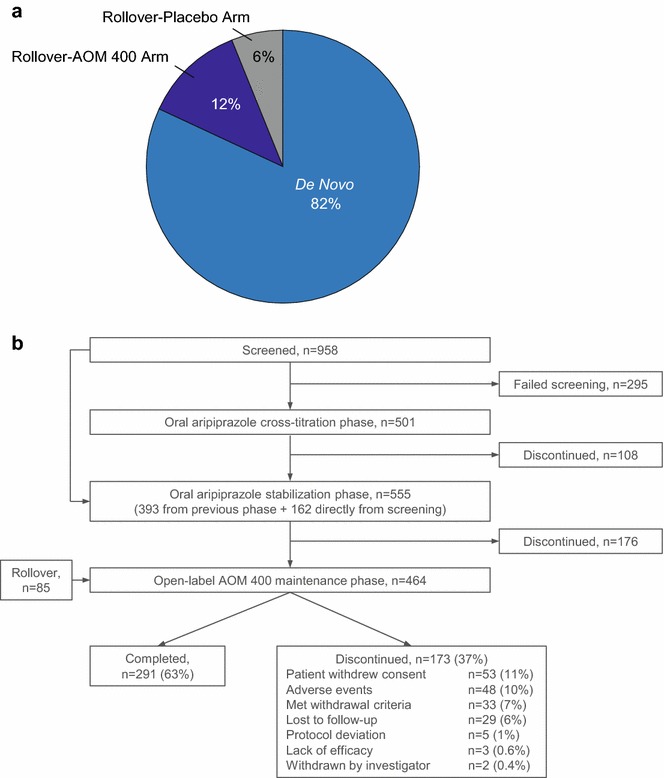
**a** Source of patients participating in this clinical trial. de novo = patients who did not participate in the lead-in study and had no prior AOM 400 exposure; Rollover-AOM 400 Arm = rollover patients from the lead-in study who had been randomized to the AOM 400 arm; Rollover-Placebo Arm = rollover patients from the lead-in study who had been randomized to the placebo arm. **b** Patient disposition across the study. Patients entered the oral aripiprazole cross-titration phase if they were not already receiving oral aripiprazole in their treatment regimen at screening. Those already on aripiprazole therapy at screening entered directly into the oral aripiprazole stabilization phase. *AOM 400* aripiprazole once-monthly 400 mg

A total of 464 patients entered the AOM 400 maintenance phase of this study. Figure 1a details the proportion of patients who were de novo (n = 379; 82%), rollover from the placebo arm of the lead-in study (n = 30; 6%), and rollover from the AOM 400 arm of the lead-in study (n = 55; 12%). This distinction is important because it indicates different pre-maintenance stabilization protocols for de novo versus rollover patients and different total AOM 400 exposure times for placebo arm versus AOM 400 arm rollover patients. De novo patients were stabilized on oral aripiprazole only (before entering the trial, or after if previously on a different oral antipsychotic), with no stabilization on AOM 400 before the AOM 400 maintenance phase of this study, while all rollover patients were stabilized for 12–28 weeks on AOM 400 according to lead-in study protocol. Rollover patients randomized to the placebo arm had no further exposure to AOM 400 before this study, while rollover patients randomized to the AOM 400 arm of the lead-in study received 52 weeks of AOM 400 during the randomized phase, resulting in a total of 64–80 weeks of exposure to AOM 400 before the 52-week maintenance phase of the current study.

Of 958 de novo patients screened, 663 met eligibility criteria. Figure 1b details patient disposition during each treatment phase of the study, and denotes the entry point of rollover patients from the lead-in trial. Of the 464 patients who entered the open-label AOM 400 maintenance phase, 379 were de novo and 85 were rollover patients. Of these, 291 (63%) completed 52 weeks of treatment. The most frequent reasons for discontinuation were withdrawal of consent (53/464; 11.4%) and AEs (48/464; 10.3%). Lack of efficacy led to study discontinuation in a small number of patients (3/464; 0.6%).

Demographic (Additional file 1: Table S1) and clinical characteristics at baseline of the open-label AOM 400 maintenance phase were similar between de novo and rollover patients. Briefly, 57.8% of patients were female, the mean age was 41.1 years, the mean BMI was 30.2 kg/m^2^, and age at first BP-I diagnosis was 29.1 years. Mean (SD) YMRS total score was 2.3 (2.9), MADRS score was 3.2 (3.2), CGI-BP-S mania score was 1.5 (0.8), CGI-BP-S depression score was 1.6 (0.8), and CGI-BP-S overall score was 1.7 (0.8).

### Safety and tolerability

The primary objective of this study was to evaluate the safety of AOM 400 administered every 4 weeks over up to 52 weeks of maintenance therapy for BP-I. During the AOM 400 maintenance phase, patients received up to 13 injections in the gluteal muscle. Injection site AEs occurred in 42 out of 464 patients (9.1%) across de novo and rollover patients, and included pain (n = 34; 7.3%), swelling (n = 4; 0.9%), bruising (n = 2; 0.4%), mass (n = 2; 0.4%), erythema (n = 1; 0.2%), and induration (n = 1; 0.2%). VAS assessment of the injections remained low, with mean patient ratings remaining at 5 or below on a scale of 0–100 throughout the maintenance phase (injections 1–13). Overall, injection site pain and reactions were minimal, decreased with subsequent injections, and were consistent with those reported in the lead-in study (Calabrese et al. 2017).

Throughout the maintenance phase, 374 of 464 patients (80.6%) experienced ≥ 1 TEAE, with 85.2% of de novo and 60.0% of rollover patients experiencing any TEAE, and 11.6% of de novo and 3.5% of rollover patients discontinuing the study owing to TEAEs (Table 1). Among AEs leading to discontinuation, bipolar I disorder, bipolar disorder, major depression, and mania were reported in 4 (0.9%), 2 (0.4%), 1 (0.2%), and 3 (0.4%) patients, respectively.

**Table 1:** Incidence of AEs during the AOM 400 maintenance phase

AE, n (%)	De novo, n = 379	Rollover, n = 85	Total, N = 464
TEAEs	323 (85.2)	51 (60.0)	374 (80.6)
Serious TEAEs	27 (7.1)	3 (3.5)	30 (6.5)
Nonserious TEAEs	318 (83.9)	49 (57.6)	367 (79.1)
Severe TEAEs	39 (10.3)	2 (2.4)	41 (8.8)
Discontinued AOM 400 due to TEAEs	44 (11.6)	3 (3.5)	47 (10.1)
Discontinued AOM 400 due to AE/death	45 (11.9)	3 (3.5)	48 (10.3)
Deaths	1 (0.3)	0	1 (0.2)

*AE* adverse event, *AOM 400* aripiprazole once-monthly 400 mg, *TEAE* treatment-emergent AE

**Fig. 2:**
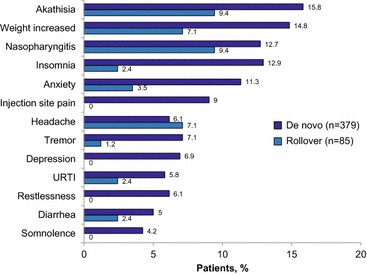
TEAEs occurring in ≥ 5% of patients during the AOM 400 maintenance phase, by enrollment source. TEAEs displayed from most common overall at top to least common overall at bottom, displayed as percent incidence in de novo and rollover patients. AOM 400 = aripiprazole once-monthly 400 mg; *TEAE* treatment-emergent adverse event, *URTI* upper respiratory tract infection

The most commonly occurring TEAEs, occurring in ≥ 5% of patients overall, are shown in Fig. 2, with the most common for de novo and rollover being akathisia (15.8% vs 9.4%), weight increase (14.8% vs 7.1%), nasopharyngitis (12.7% vs 9.4%), and insomnia (12.9% vs 2.4%). Mean (SD) change in weight from baseline to the last visit of the AOM 400 maintenance phase was 0.5 (7.0) kg for de novo patients and 0.6 (4.8) kg for rollover patients. Potentially clinically relevant weight gain (≥ 7%) at the last visit of the maintenance phase was observed in 61/370 de novo patients (16.5%) and 10/84 rollover patients (11.9%), while potentially clinically relevant weight loss was observed in 42 de novo patients (11.4%) and 6 rollover patients (7.1%). Overall, the incidence of TEAEs was low, and TEAEs occurred early and did not appear to increase with time on treatment. Mean changes from baseline in extrapyramidal symptoms, as assessed by specific rating scales, were minimal and not clinically meaningful (Additional file 2: Table S2). No clinically meaningful changes in clinical laboratory parameters were observed.

Treatment-emergent adverse events related to suicidal ideation/suicide were reported in 11/464 patients (2.4%) during the maintenance phase, with the events considered serious in 4 patients. Treatment-emergent possible suicidal events assessed using the C-SSRS were reported in 46/464 patients (9.9%), including suicidal ideation (9.9%), non-suicidal self-injurious behavior (0.9%), suicide attempt (0.6%), and preparatory action toward imminent suicidal behavior (0.9%).

Although serious AEs (SAEs) were rare, 27 de novo (7.1%) and 3 rollover (3.5%) patients reported SAEs during maintenance treatment with AOM 400, including 1 death in the de novo cohort (acute myocardial infarction) that was not considered related to study drug. SAEs considered related to study drug included BP-I (2 patients) and diabetes mellitus, obesity, somnolence, tardive dyskinesia, bipolar disorder, major depression, mania, and dyspnea (1 patient each).

### Efficacy

**Fig. 3:**
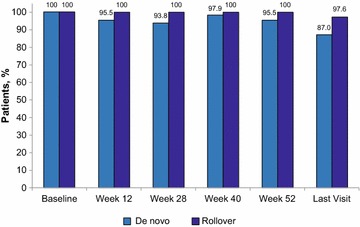
Percentage of patients remaining stable throughout the AOM 400 maintenance phase, by enrollment source. Stability defined as (1) outpatient status, (2) Young Mania Rating Scale total score ≤ 12, (3) MADRS total score ≤ 12, and (4) no active suicidality, with active suicidality defined as MADRS item 10 score ≥ 4, or “yes” on question 4 or 5 of the Columbia Suicide Severity Rating Scale. *MADRS* Montgomery–Asberg Depression Rating Scale, *n* number evaluable

The secondary objective of this study was to evaluate the long-term therapeutic effect of AOM 400 as maintenance treatment for BP-I in de novo and rollover patients. Efficacy was measured first as the proportion of patients stable at baseline (beginning of the AOM 400 maintenance phase) who remained stable at the end of AOM 400 maintenance treatment (Fig. 3). Overall, 89% of patients who were stable at baseline were stable at their last visit of the maintenance treatment phase.

Efficacy was also measured by assessing the change in key clinical stability indicators, including YMRS total score, MADRS total score, CGI-BP-S overall score, CGI-BP-S mania score, and CGI-BP-S depression score, as outlined in Table 2. The mean YMRS and CGI-BP-S (overall, mania, and depression) scores were largely unchanged, with slight improvement from baseline.

**Table 2:** Change in clinical stability indicators during the AOM 400 maintenance phase

Mean (SD) change from baseline to last visit (up to week 52; LOCF)						
Assessment	De novo		Rollover		Total	
	N	Result	N	Result	N	Result
YMRS total score	377	− 0.33 (3.87)	85	− 0.16 (4.42)	462	− 0.30 (3.97)
MADRS total score	377	1.36 (6.93)	85	0.67 (3.16)	462	1.24 (6.41)
CGI-BP-S overall score	353	0.13 (1.20)	84	− 0.01 (0.69)	437	0.10 (1.12)
CGI-BP-S mania score	353	− 0.07 (0.83)	84	− 0.08 (0.71)	437	− 0.07 (0.81)
CGI-BP-S depression score	353	0.18 (1.14)	84	0.12 (0.52)	437	0.17 (1.05)

*AOM 400* aripiprazole once-monthly 400 mg, *CGI-BP-S* Clinical Global Impressions for Bipolar Disorder-Severity of Illness Scale, *LOCF* last observation carried forward, *MADRS* Montgomery**–**Asberg Depression Rating Scale, *SD* standard deviation, *YMRS* Young Mania Rating Scale

Use of rescue medication during the AOM 400 maintenance phase is shown in Table 3. Less than 8% of patients used lithium (6.0%) or valproate (7.8%), all but one of whom were de novo patients.

**Table 3:** Rescue medication use during the AOM 400 maintenance phase

Medication	Enrollment source	Number of patients	Number (%) taking rescue medication	Duration (days)^a^		
				Mean	SD	Median
Lithium	De novo	379	28 (7.4)	147.9	101.8	131.5
	Rollover	85	0	–	–	–
	Total	464	28 (6.0)	147.9	101.8	131.5
Valproate	De novo	379	35 (9.2)	114.5	96.6	100
	Rollover	85	1 (1.2)	84.0	–	84.0
	Total	464	36 (7.8)	113.7	95.4	92.0

Rescue medication (lithium [immediate or controlled release] or valproate [immediate or extended release]) was recommended for patients not meeting stability criteria. Only one type of rescue medication was permitted at a time. Patients meeting stability criteria were not eligible for rescue medication

*AOM 400* aripiprazole once-monthly 400 mg, *SD* standard deviation

^a^Calculated for patients receiving AOM 400 during maintenance phase who took rescue medication during maintenance phase

### Patient satisfaction and patient-assessed tolerability

**Fig. 4:**
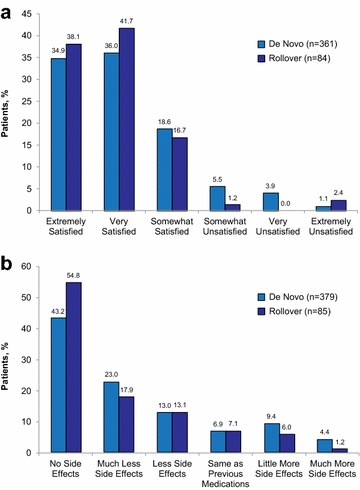
Patient evaluation of own satisfaction with AOM 400 (**a**) and patient assessment of side effects on AOM 400 relative to previous medication (**b**) using the Patient Satisfaction with Medication Questionnaire-Modified; data collected at last visit of the AOM 400 maintenance phase. *AOM 400* aripiprazole once-monthly 400 mg

An additional secondary objective was to assess the acceptability and long-term tolerability of AOM 400 from a patient perspective, using qualitative patient satisfaction and side effect profiles as evaluation measures. Using the PSMQ-M, patients could directly share their experience of taking AOM 400 with clinicians. Figure 4a, b show that > 70% of patients in either group were extremely or very satisfied with AOM 400 treatment, and > 65% had either no side effects or many fewer side effects than with previous medications.

## Discussion

Overall, these data demonstrate the safety and efficacy of AOM 400 for the maintenance treatment of BP-I. The finding that, after switching from a previous antipsychotic, patients stable on oral aripiprazole can be safely switched to AOM 400 is important because it opens the use of AOM 400 for maintenance treatment by patients in the clinic without the need for lengthy pre-stabilization protocols. Making the switch to AOM 400 for long-term maintenance may prove to be a simple and effective clinical next step. With maintained efficacy and a good tolerability profile, patient satisfaction was high, suggesting AOM 400 may be well accepted with broader clinical use.

This study, combined with the previously published lead-in study (Calabrese et al. 2017), provides robust data on the only once-monthly LAI atypical antipsychotic approved by the FDA for maintenance monotherapy of BP-I (Citrome 2017). The 2 cohorts of patients included in this study (de novo and rollover) exhibited similar maintenance phase outcomes, including low discontinuation rates, low need for rescue medications, high rates of stability, and high levels of patient-assessed satisfaction relative to previous medications. Rollover patients provide the longest maintenance treatment period data for analysis, and de novo patients represent a real-world practice setting, in which patients on other treatments could be effectively switched to AOM 400. Because LAI formulations of antipsychotics have been shown to reduce relapse rates and hospitalizations, and improve brain health and long-term societal functioning (Kishimoto et al. 2013; Subotnik et al. 2015), a more general shift from oral to LAI aripiprazole may be justified for eligible patients.

The majority of randomized controlled trials that have been performed to assess the efficacy of SGAs in patients with bipolar disorder are enriched for responders (Lindström et al. 2017); in contrast, this trial cohort was not. The majority of participants in this study (82%) were classified as de novo patients, meaning they were not stabilized on AOM 400 before being maintained on AOM 400. Instead, these patients were stabilized on oral aripiprazole and then switched to AOM 400 for maintenance. As a result, the high percentage of de novo patients with stable disease seen in our study is not due to selection bias; instead, it reflects the efficacy of AOM 400 for maintenance treatment of BP-I in a population of patients and a clinical scenario more closely representing those routinely encountered in clinical practice. Furthermore, the rate of study discontinuation due to lack of treatment efficacy was very low, and overall study completion rate was high. Notably, de novo patients demonstrated a low rate of discontinuation due to TEAEs and expressed high levels of satisfaction with the medication, suggesting a safety and tolerability profile similar to that seen in the earlier randomized withdrawal study (Calabrese et al. 2017).

The remaining 18% of the study population consisted of patients previously stabilized on AOM 400, and thus represent a subset of respondent-enriched participants (Calabrese et al. 2017). All TEAEs were less common in rollover versus de novo patients, which might be due to the enriched population. It could also reflect a tendency for some TEAEs to emerge earlier in the treatment course. As with de novo patients, a large proportion of rollover patients reported high satisfaction with their treatment and less bothersome side effects versus their previous medication. To be eligible to rollover into this study, these participants had to have completed the full trial mentioned above, which for some individuals resulted in exposure to AOM 400 exceeding 2 years (up to 2.5 years) across the 2 studies.

The 2 notable aspects of this trial, the relative lack of enrichment design (because the patients did not require prior stabilization on AOM 400) and the permitted use of rescue medications, allow the results to more closely reflect what may be seen if AOM 400 were to be used for maintenance treatment of BP-I in the clinic. In most cases, healthcare providers may choose to use oral antipsychotics for management of acute mood crises, while LAI forms may be preferred for long-term maintenance in an outpatient setting (Citrome 2017).

The efficacy of AOM 400 as maintenance treatment in reducing the occurrence of mood episodes in BP-I has been reported previously (Calabrese et al. 2017); however, data on relapse rates was not collected in this study, limiting the ability to directly compare the effectiveness of long-term AOM 400 maintenance treatment for BP-I with other similar studies in long-term treatment settings.

## Conclusion

To our knowledge, this is the longest recorded evidence of safety and efficacy of any formulation of an atypical LAI antipsychotic and the only one approved for monthly injection for the maintenance treatment of BP-I. We have shown long-term safety and efficacy in a large data set in which a majority of patients maintained stability through 52 weeks of AOM 400 treatment with minimal need for rescue medication. These data support the broader use of AOM 400 for maintenance treatment of BP-I.

## Additional files

**Additional file 1: Table S1.** Demographic characteristics of study population.

**Additional file 2: Table S2.** Change from baseline to week 52 in extrapyramidal symptoms scales during AOM 400 maintenance phase.

## Abbreviations

AE: adverse event
AOM 400: aripiprazole once-monthly 400 mg
BP-I: bipolar I disorder
CGI-BP-S: Clinical Global Impressions for Bipolar Disorder-Severity of Illness Scale
C-SSRS: Columbia Suicide Severity Rating Scale
ECG: electrocardiogram
LAI: long-acting injectable
MADRS: Montgomery–Asberg Depression Rating Scale
PSMQ-M: Patient Satisfaction with Medication Questionnaire-Modified
SAE: serious adverse event
SGA: second-generation antipsychotic
TEAE: treatment-emergent adverse event
VAS: Visual Analog Scale
YMRS: Young Mania Rating Scale
