# Supplementary material for: Aripiprazole once-monthly as maintenance treatment for bipolar I disorder: a 52-week, multicenter, open-label study
Source: Int J Bipolar Disord. 2018 Jun 10;6:14. doi: 10.1186/s40345-018-0122-z (PMC6162003; doi:10.1186/s40345-018-0122-z)
Supplement: Supplementary file 1 — Additional file 1: Table S1. Demographic characteristics of study population. [file 40345_2018_122_MOESM1_ESM.docx]

## Table S1. Demographic Characteristics of Study Population

| **Characteristic** | ***De Novo***  **(n=379)** | **Rollover**  **(n=85)** | **Total**  **(N=464)** |
| --- | --- | --- | --- |
| Sex, n (%) | | | |
| Male | 159 (42.0) | 37 (43.5) | 196 (42.2) |
| Female | 220 (58.0) | 48 (56.5) | 268 (57.8) |
| Race, n (%) | | | |
| White | 216 (57.0) | 39 (45.9) | 255 (55.0) |
| Black/African American | 77 (20.3) | 27 (31.8) | 104 (22.2) |
| Asian | 78 (20.6) | 16 (18.8) | 94 (20.3) |
| Other | 8 (2.1) | 3 (3.5) | 11 (2.4) |
| Ethnicity, n (%) | | | |
| Hispanic or Latino | 24 (6.3) | 5 (5.9) | 29 (6.3) |
| Non-Hispanic or Latino | 355 (93.7) | 80 (94.1) | 435 (93.8) |
| Region, n (%) |  |  |  |
| North America | 265 (69.9) | 60 (70.6) | 325 (70.0) |
| Europe | 39 (10.3) | 8 (9.4) | 47 (10.1) |
| Japan | 65 (17.2) | 10 (11.8) | 75 (16.2) |
| Other Asian countries | 10 (2.6) | 7 (8.2) | 17 (3.7) |
| Age (y), mean (SD) | 41.0 (12.0) | 41.6 (10.7) | 41.1 (11.8) |
| Weight (kg), mean (SD) | 85.9 (24.7) | 89.5 (25.5) | 86.6 (24.8) |
| Body mass index (kg/m^2^), mean (SD) | 30.0 (7.7) | 30.9 (8.1) | 30.2 (7.7) |

SD=standard deviation.
