# Supplementary material for: Aripiprazole once-monthly as maintenance treatment for bipolar I disorder: a 52-week, multicenter, open-label study
Source: Int J Bipolar Disord. 2018 Jun 10;6:14. doi: 10.1186/s40345-018-0122-z (PMC6162003; doi:10.1186/s40345-018-0122-z)
Supplement: Supplementary file 2 — Additional file 2: Table S2. Change from baseline to week 52 in extrapyramidal symptoms scales during AOM 400 maintenance phase. [file 40345_2018_122_MOESM2_ESM.docx]

**Table S2. Change From Baseline to Week 52^a^ in Extrapyramidal Symptoms Scales During AOM 400 Maintenance Phase**

| **Scale, mean (SD)** | ***De Novo*** | **Rollover** | **Total** |
| --- | --- | --- | --- |
| Simpson-Angus Scale total score^b^ | 0.21 (1.65) | 0.17 (1.25) | 0.20 (1.58) |
| Drug-Induced Extrapyramidal Symptoms Scale total score^b^ | 0.22 (1.17) | 0.20 (0.63) | 0.21 (1.11) |
| Barnes Akathisia Rating Scale global score | 0.04 (0.62) | 0.02 (0.51) | 0.04 (0.60) |
| Abnormal Involuntary Movement Scale rating score | 0.06 (1.07) | 0.01 (0.19) | 0.05 (0.97) |

AOM 400=aripiprazole once-monthly 400 mg; SD=standard deviation.

^a^Last observation carried forward.

^b^Simpson-Angus Scale was used at non-Japanese sites; Drug-Induced Extrapyramidal Symptoms Scale was used at Japanese site.
